# Innovative application of a cystotome in the recanalization of a tight pancreatic duct stricture for abscess drainage

**DOI:** 10.1055/a-2463-5860

**Published:** 2024-12-10

**Authors:** Ping Yue, Chenjun Tian, Yanxian Ren, Jinduo Zhang, Wenbo Meng, Xun Li

**Affiliations:** 1 Department of General Surgery, The First Hospital of Lanzhou University, Lanzhou, China; 2 The First School of Clinical Medicine, Lanzhou University, Lanzhou, China; 3 Hepatopancreatobiliary Surgery Institute of Gansu Province, Lanzhou, China

A 65-year-old man was hospitalized with left upper abdominal pain and fever. Computed tomography showed an abscess in the pancreatic tail, and magnetic resonance cholangiopancreatography also confirmed multiple pancreatic duct strictures ( Fig. 1 ). The patient had experienced acute severe pancreatitis 5 years previously and had also undergone placement of two coronary stents 1 month earlier because of acute myocardial infarction.

**Fig. 1:**
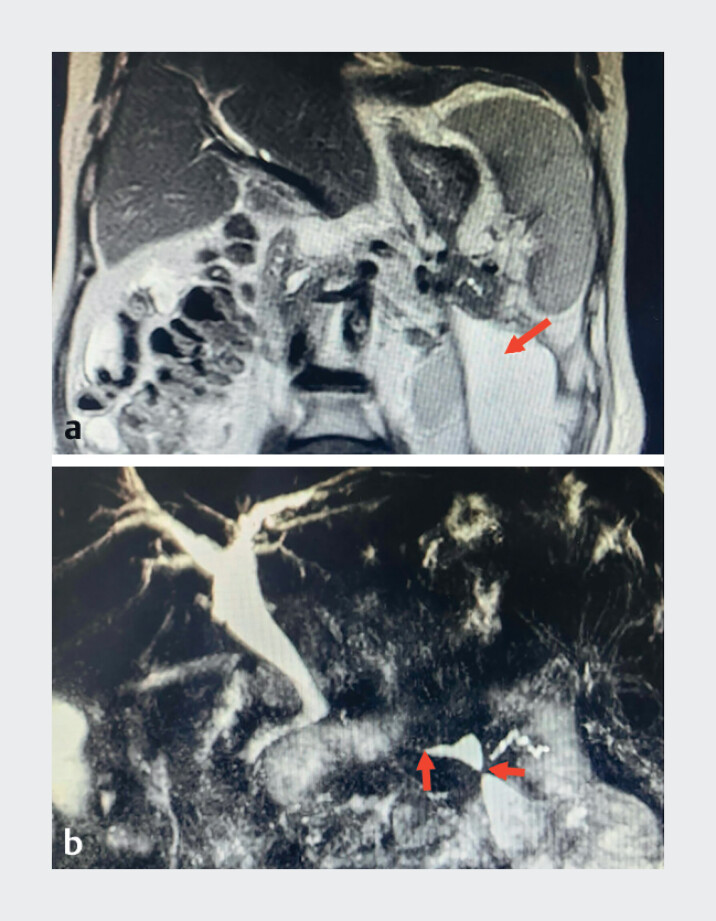
Imaging studies. **a** Magnetic resonance imaging showed an abscess in the pancreatic tail (red arrow). **b** Magnetic resonance cholangiopancreatography confirmed multiple pancreatic duct strictures (red arrow).

As the abscess was far from the gastric wall, endoscopic retrograde cholangiopancreatography (ERCP) drainage was selected instead of endoscopic ultrasonography (EUS). The pancreatic duct was successfully cannulated, but the 0.025-inch guidewire failed to pass through the strictures, and the contrast agent could not enter the distal pancreatic duct ( Fig. 2 ). The guidewire was replaced with a loach guidewire (0.035 inches in diameter, 260 cm in length; Terumo Vietnam, Hanoi, Vietnam), which could cross the stenosis into the abscess; however, the accessories, including a Soehendra biliary dilation catheter (Cook Medical, Winston-Salem, North Carolina, USA) could not pass through the stricture despite repeated attempts ( Fig. 3 ). Therefore, electroincision with a 6-Fr cystotome (Cysto-Gastro-Set SU; ENDO-FLEX GmbH, Voerde, Germany) was used to recanalize the stricture successfully ( Fig. 4 ), and a nasal-pancreatic abscess drainage tube was placed after copious pus was aspirated ( Fig. 5 , Video 1 ). The amylase level in the pus was 39520 U/L (normal <150 U/L). A plastic stent was placed during repeat ERCP 3 days later when the abscess had disappeared. The patient recovered rapidly and uneventfully after the ERCP procedures.

**Fig. 2:**
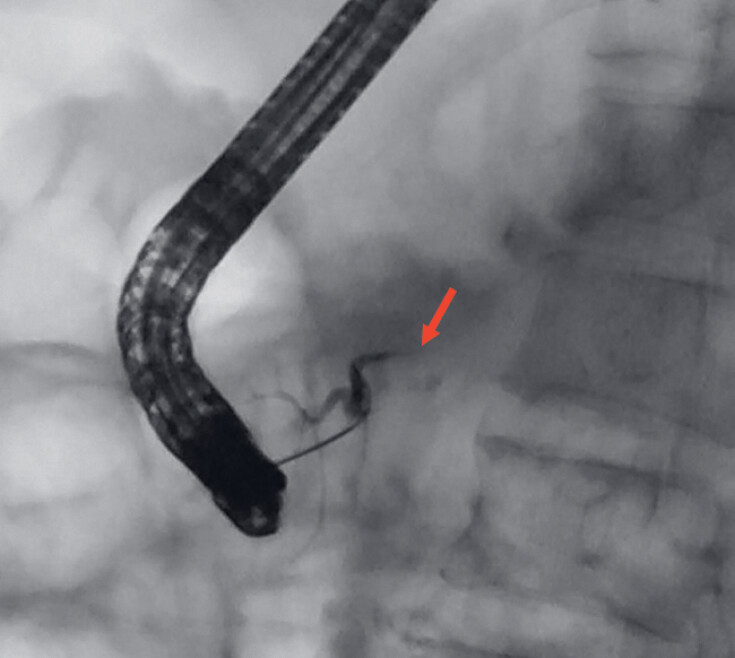
Fluoroscopy showed that the guidewire failed to pass through the strictures despite repeated attempts, and the contrast agent could not enter the distal pancreatic duct (red arrow).

**Fig. 3:**
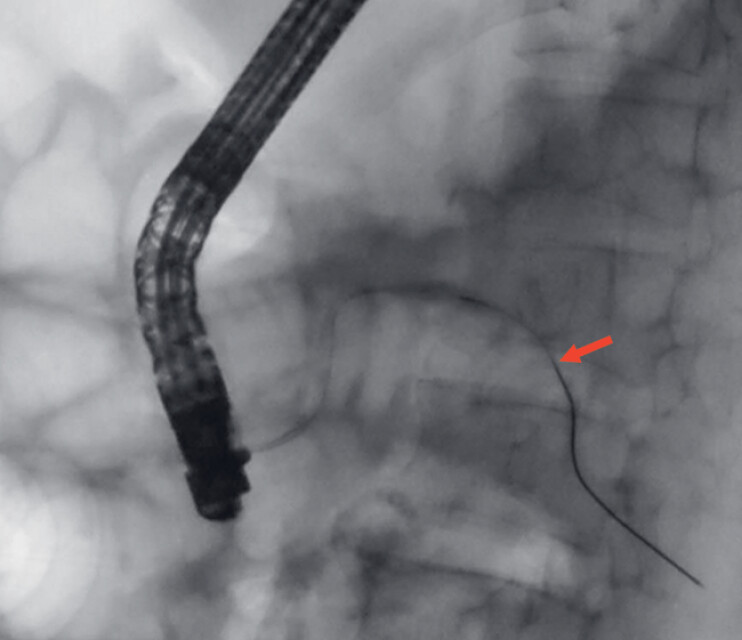
A loach guidewire crossed the stenosis into the abscess (red arrow).

**Fig. 4:**
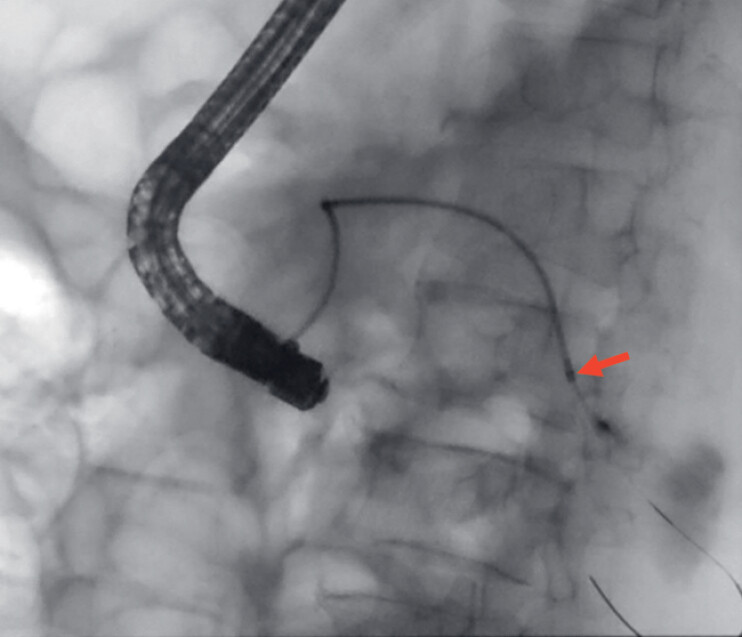
Electroincision with a 6-Fr cystotome recanalized the strictures successfully (red arrow).

**Fig. 5:**
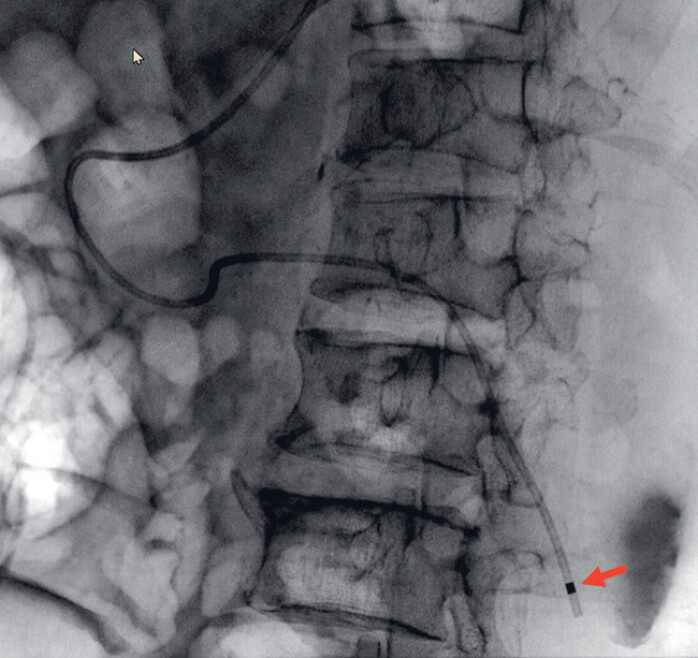
A nasal-pancreatic drainage tube was placed (red arrow).

Endoscopic retrograde cholangiopancreatography for recanalization of the pancreatic duct and drainage of the abscess in the pancreatic tail.Video 1

For pancreatic pseudocysts communicating with the pancreatic duct and located some distance from the stomach, EUS and percutaneous drainage are unsuitable and risk creating a persistent pancreatic fistula. Instead, internal drainage by ERCP is the preferred option 1 , but pancreatic duct stenosis hinders its technical success. Tringali et al. 2 reported the incision of a pancreatic duct stricture using a needle-knife. In the current case, a cystotome with pure cut mode was used to recanalize multiple pancreatic duct strictures safely and effectively. To our knowledge, this is the first report of this procedure, which provides a new option for similar cases.

Endoscopy_UCTN_Code_TTT_1AR_2AI

## Notes

Endoscopy E-Videos https://eref.thieme.de/e-videos
*E-Videos* is an open access online section of the journal *Endoscopy* , reporting on interesting cases and new techniques in gastroenterological endoscopy. All papers include a high-quality video and are published with a Creative Commons CC-BY license. Endoscopy E-Videos qualify for HINARI discounts and waivers and eligibility is automatically checked during the submission process. We grant 100% waivers to articles whose corresponding authors are based in Group A countries and 50% waivers to those who are based in Group B countries as classified by Research4Life (see: https://www.research4life.org/access/eligibility/ ). This section has its own submission website at https://mc.manuscriptcentral.com/e-videos .
